# Screening programs for renal cell carcinoma: a systematic review by the EAU young academic urologists renal cancer working group

**DOI:** 10.1007/s00345-022-03993-6

**Published:** 2022-04-01

**Authors:** Pietro Diana, Tobias Klatte, Daniele Amparore, Riccardo Bertolo, Umberto Carbonara, Selcuk Erdem, Alexandre Ingels, Onder Kara, Laura Marandino, Michele Marchioni, Stijn Muselaers, Nicola Pavan, Angela Pecoraro, Alessio Pecoraro, Eduard Roussel, Riccardo Campi, the European Association of Urology (EAU) Young Academic Urologists (YAU) Renal Cancer group

**Affiliations:** 1 Department of Urology, Fundació Puigvert, Autonoma University of Barcelona, Cartagena 340-350, 08025 Barcelona, Spain; 2 Department of Urology, Humanitas Clinical and Research Institute IRCCS, Rozzano, Italy; 3 Department of Urology, Charité-Universitätsmedizin Berlin, Berlin, Germany; 4 Division of Urology, Department of Oncology, School of Medicine, San Luigi Hospital, University of Turin, Orbassano, Turin, Italy; 5 European Association of Urology (EAU) Young Academic Urologists (YAU) Renal Cancer Working Group, Arnhem, The Netherlands; 6 Department of Urology, San Carlo Di Nancy Hospital, Rome, Italy; 7 Department of Emergency and Organ Transplantation-Urology, Andrology and Kidney Transplantation Unit, University of Bari, Bari, Italy; 8 Division of Urologic Oncology, Department of Urology, Istanbul University Istanbul Faculty of Medicine, Istanbul, Turkey; 9 Department of Urology, University Hospital Henri Mondor, APHP, 51 Avenue du Maréchal de Lattre de Tassigny, 94010 Créteil, France; 10 Biomaps, UMR1281, INSERM, CNRS, CEA, Université Paris Saclay, Villejuif, France; 11 Department of Urology, Kocaeli University School of Medicine, Izmit, Turkey; 12 Department of Medical Oncology, IRCCS Ospedale San Raffaele, Vita-Salute San Raffaele University, Milan, Italy; 13 Department of Medical, Oral and Biotechnological Sciences, Laboratory of Biostatistics, University “G. D’Annunzio” Chieti-Pescara, Chieti, Italy; 14 Department of Urology, SS Annunziata Hospital, “G. D’Annunzio” University of Chieti, Chieti, Italy; 15 Department of Urology, Radboud University Medical Center, Nijmegen, The Netherlands; 16 Urology Clinic, Department of Medical, Surgical and Health Science, University of Trieste, Trieste, Italy; 17 Unit of Urological Robotic Surgery and Renal Transplantation, Careggi Hospital, University of Florence, Florence, Italy; 18 Department of Urology, University Hospitals Leuven, Leuven, Belgium; 19 Department of Experimental and Clinical Medicine, University of Florence, Florence, Italy

**Keywords:** Renal cancer, Kidney, Cancer, Screening, Imaging, Biomarkers

## Abstract

**Purpose:**

To systematically review studies focused on screening programs for renal cell carcinoma (RCC) and provide an exhaustive overview on their clinical impact, potential benefits, and harms.

**Methods:**

A systematic review of the recent English-language literature was conducted according to the European Association of Urology guidelines and the PRISMA statement recommendations (PROSPERO ID: CRD42021283136) using the MEDLINE, Cochrane Central Register of Controlled Trials, and ClinicalTrials.gov databases. Risk-of-bias assessment was performed according to the QUality In Prognosis Studies (QUIPS) tool.

**Results:**

Overall, nine studies and one clinical trials were included. Eight studies reported results from RCC screening programs involving a total of 159 136 patients and four studies reported screening cost-analysis. The prevalence of RCC ranged between 0.02 and 0.22% and it was associated with the socio-demographic characteristics of the subjects; selection of the target population decreased, overall, the screening cost per diagnosis.

**Conclusions:**

Despite an increasing interest in RCC screening programs from patients and clinicians there is a relative lack of studies reporting the efficacy, cost-effectiveness, and the optimal modality for RCC screening. Targeting high-risk individuals and/or combining detection of RCC with other health checks represent pragmatic options to improve the cost-effectiveness and reduce the potential harms of RCC screening.

**Supplementary Information:**

The online version contains supplementary material available at 10.1007/s00345-022-03993-6.

## Introduction

The incidence of renal cell carcinoma (RCC) is increasing, with over 430.000 new cases diagnosed per year and over 175.000 new deaths per year in 2020 worldwide [1]. Risk factors for RCC are well established and include older age, male sex, smoking, hypertension, and obesity [2]. The persistent increase in incidence is most likely related to both an increase in risk factors prevalence in the society and to a higher incidence in incidental detection [3–5]; such epidemiological signature confirms the unmet need to reduce overdiagnosis and overtreatment in the future [6].

Although most RCCs are diagnosed at an early stage in asymptomatic patients [7] and up to 25% present with metastasis showing a 1-year and 5-year survival of 39% and 12% versus 96% and 86% in patients diagnosed with a stage I disease [8]. These figures make RCC one of the deadliest genitourinary tumors [9]. Moreover, if diagnosed at an advanced stage, RCC is not likely curable by surgery alone, increasing the complexity and costs of treatment, as well as a non-negligible risk of adverse events, with worse patient-reported outcomes.

Taken together, the increasing prevalence of RCC risk factors in the general population [10], coupled with a high proportion of asymptomatic patients and a high mortality rate make RCC suitable for screening programs. The ultimate objective of such programs should be to increase the detection of early stage tumors that deserve timely treatment, to improve survival outcomes, the patient’s quality of life, and to decrease healthcare costs, toward the concept of value-based healthcare [11].

Focused renal ultrasound (US) has been reported to be a cost-effective tool with a potential survival benefit associated with early detection through RCC screening [12, 13]. In addition, several liquid biomarkers, focusing on genetic or metabolic assays, and innovative imaging tools, have been recently tested for the detection of RCC [14]. Despite being attractive non-invasive diagnostic modalities, none of them have been validated or shown to have clinical utility to be implemented in the daily practice [14].

Overall, screening programs targeting high-risk individuals have the potential to be cost-effective strategies to improve RCC care. However, several uncertainties still remain, including the magnitude of the benefit of early treatment, the overall cost-effectiveness of the screening, the optimal screening modality and target population, as well as the potential harms of screening programs [15]. In fact, a possible drawback of any screening program is the risk of overdiagnosis, psychological distress, and financial toxicity for both individual patients and the whole society [12, 15].

The aim of this systematic review is to summarize the current evidence on available screening programs for RCC, focusing on their potential benefits, harms, and impact on current and future clinical practice.

## Materials and methods

### Evidence acquisition

This systematic review was conducted according to the principles highlighted by European Association of Urology (EAU) Guidelines Office [16] and the updated Preferred Reporting Items for Systematic Reviews and Meta-analyses (PRISMA) recommendations [17].

### Review protocol

The methods for this systematic review were summarized following the PRISMA for Protocols 2015 (PRISMA-P 2015) statement recommendations (www.equator-network.org/reporting-guidelines/prisma-protocols/) [18]. The protocol was registered in the International Prospective Register of Ongoing Systematic Reviews (PROSPERO, http://www.crd.york.ac.uk/prospero) on December 20th, 2021 (registration ID: CRD42021283136).

### Search strategy

A systematic review of the English-language literature was performed according to the PRISMA criteria [17] using the Medline, Web of Science and Embase databases in November 2021. In addition, ClinicalTrials.gov was searched on December 15th, 2021 for potential clinical trials on RCC screening.

The literature search used both free text and MeSH terms (keywords: “screening” OR “cancer screening” AND “renal cell carcinoma” OR “renal neoplasm” OR “renal cancer” OR “kidney cancer” OR “kidney neoplasm”). A detailed overview of the search strategy is available in the Appendix. The search strategy was adapted for the databases other than MEDLINE, as appropriate. An updated search was performed on December 15th, 2021 to identify additional relevant records. A manual search of bibliographies from included studies and previous systematic reviews was also performed.

### Eligibility criteria

A specific population (P), intervention (I), comparator (C), and outcome (O) framework defined the study eligibility, as recommended [16, 19]. In brief, studies were considered eligible if they fulfilled the following criteria:

- (P): adult (> 18 years) healthy subjects or adult individuals at higher risk of developing renal cell carcinoma (RCC) based on established risk factors (age, male gender, family history, smoking, obesity, diabetes, hypertension) with no prior history of RCC (or prior imaging showing a renal mass) and no prior history of diseases increasing the risk of RCC (including genetic syndromes);
- (I): any screening intervention (opportunistic or population screening), including any type of medical test (liquid biomarkers, non-invasive imaging, renal biopsy);
- (C): either comparative or non-comparative studies;
- (O): cost-effectiveness of the screening program (detection rate of histologically confirmed RCC vs costs of the screening program).
- Studies assessing the impact of screening programs on the detection of renal masses of *undetermined nature* (with no histopathological data) were excluded. Studies with insufficient reporting of the PICOS criteria were also excluded.

### Study selection

**Fig. 1:**
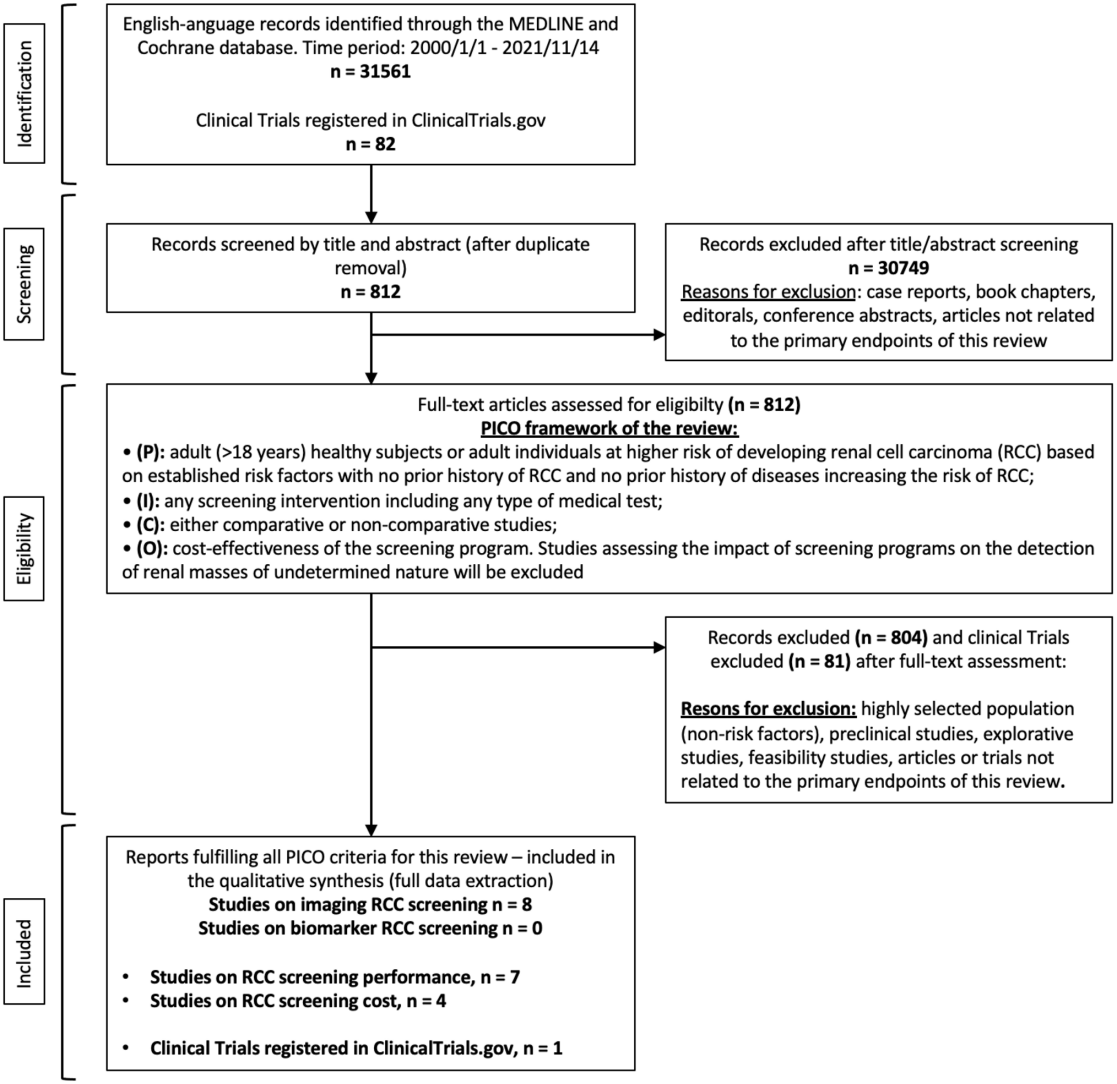
Flowchart showing the main steps of the review process and the final number of studies included and excluded according to the Preferred Reporting Items for Systematic Reviews and Meta-analyses (PRISMA) statement recommendations

Mendeley reference software removed duplicate records identified. The title and abstract of all retrieved records were screened independently by two review authors to identify records reporting the use of biomarkers or imaging tests for screening of RCC. Disagreement was solved by a third party, who supervised the review process. The list of articles judged highly relevant was reviewed by all co-authors until final consensus was reached. Three independent review authors checked the study eligibility after full-text assessment. Separate screening forms were created for each selection phase. Disagreement was solved by a third party. The records not meeting the PICO framework of this review were finally excluded. The flowchart depicting the overall review process according to the PRISMA statement recommendations [19] is shown in Fig. 1.

### Data collection and risk of bias assessment

Data from the studies included in the review were extracted by two authors in a a-priori developed data extraction form. The reliability and completeness of data extraction was crosschecked by another member of the review team. When more than one article was based on the same study population, we included the most recent report.

The same authors independently performed a formal risk of bias assessment using the QUality In Prognosis Studies (QUIPS) tool [20]. A third reviewer acted as an arbitrator. The QUIPS tool provides a measure of the risk of bias over six domains of interest (Supplementary Table 1).

The overall quality of evidence was assessed according to Grading of Recommendations Assessment, Development, and Evaluation (GRADE) recommendations (https://www.gradeworkinggroup.org; www.handbook.cochrane.org).

A narrative format was used for evidence synthesis. Due to the quality and heterogeneity of the included studies, a quantitative synthesis of the evidence was not performed.

## Results

### Study selection

The literature search identified 31,561 records and 82 clinical trials. Of these, 30,749 studies were excluded by title and abstract screening, with a further 797 and 81 clinical trials excluded after full-text assessment. Of the records screened, 9 studies and one clinical trial focused on RCC screening and fulfilling all the PICO criteria and were, therefore, included in the qualitative analysis (Table 1). Only four studies (Table 2) analyzed the costs of a RCC screening program. The study selection process is summarized in the PRISMA flowchart (Fig. 1).

**Table 1:** Studies reporting the results of the RCC screening programs

First author	Leading Country	Number of centers	Data collection dates	Type of screening	Target population	Sample size	% Male/Female	Mean age (range)	Screening modality	*N* (%) patient with suspicion of RCC	*N* (%) of RCC cases	*N* (%) of RCC detected
Ono	Japan	Single center	2003–2004	Screening for RCC	Asymptomatic	3426	58.8%/41.2%	56.4 (22–87)	FDG–PET	1 (0.03%)	3 (0.09%)	1 (0.03%)
Malaeb	USA	Multi-institutional	1993–1997	Screening for RCC	Asymptomatic (conjunction with AAA screening)	6678	97%/3%	66.2 (56–79)	US	817 (12.23%)	15 (0.22%)	15 (0.22%)
Mizuma	Japan	Multi-institutional	1990–1995	Screening for RCC	Asymptomatic	16,024	57.8%/42.2%	47 (25–84)	US	111 (0.69%)	6 (0.04%)	5 (0.03%)
Haliloglu	Turkey	Single center	1995–2008	Screening for RCC	Asymptomatic or LUTS	18,203	64%/35.9%	55 (33–90)	US	81 (0.44%)	36 (0.02%)	36 (0.02%)
Tsuboi	Japan	Single center	1993–1997	Screening for RCC	Asymptomatic	60,604	67%/33%	NR (15–96)	US	97 (0.16%)	38 (0.06%)	13 (0.02%)
Filipas	Germany	Single center	1996–1998	Screening for RCC	Asymptomatic (> 40 years)	9959	49%/51%	61 (40–94)	US	13 (0.13%)	11 (0.11%)	11 (0.11%)
Mitchell	USA	Single center	1995–1999	Screening for RCC	Asymptomatic	11,932	64%/36%	NR (34–74)	CT	27 (0.23%)	26 (0.22%)	22 (0.18%)
Feldstein	USA	Single center	2002–2007	Screening for RCC	Asymptomatic	32,310	NR	NR	CT, US, dipstick	13 (0.04%)	18 (0.06%)	13 (0.04%)

RCC, renal cell carcinoma; CT, computed tomography; US, ultrasound; PET, positron emitted tomography; NR, not reported

**Table 2:** Studies considering cost-analysis in RCC screening programs

First author	Country	Number of centers	Target population	Sample size	Screening tool	RCC cases (*n*; *x*/10^5^)	Cost per screening tool	Costs (mean) per one RCC diagnosis
Malaeb	USA	Multi-institutional	Asymptomatic (conjunction with AAA screening)	6678	US	22 (300)	107$	32,480
Mizuma	Japan	Multi-institutional	Asymptomatic	16,024	US	6 (40)	200$	671,440
Rossi	UK	Multi-institutional	Asymptomatic	NR	US	NR	38£	Detailed per age groups
Filipas	Germany	Single center	Asymptomatic (> 40 years)	9959	US	9 (9)	6$	NR

RCC, renal cell carcinoma; NR, not reported

### Study characteristics and risk of bias

**Fig. 2:**
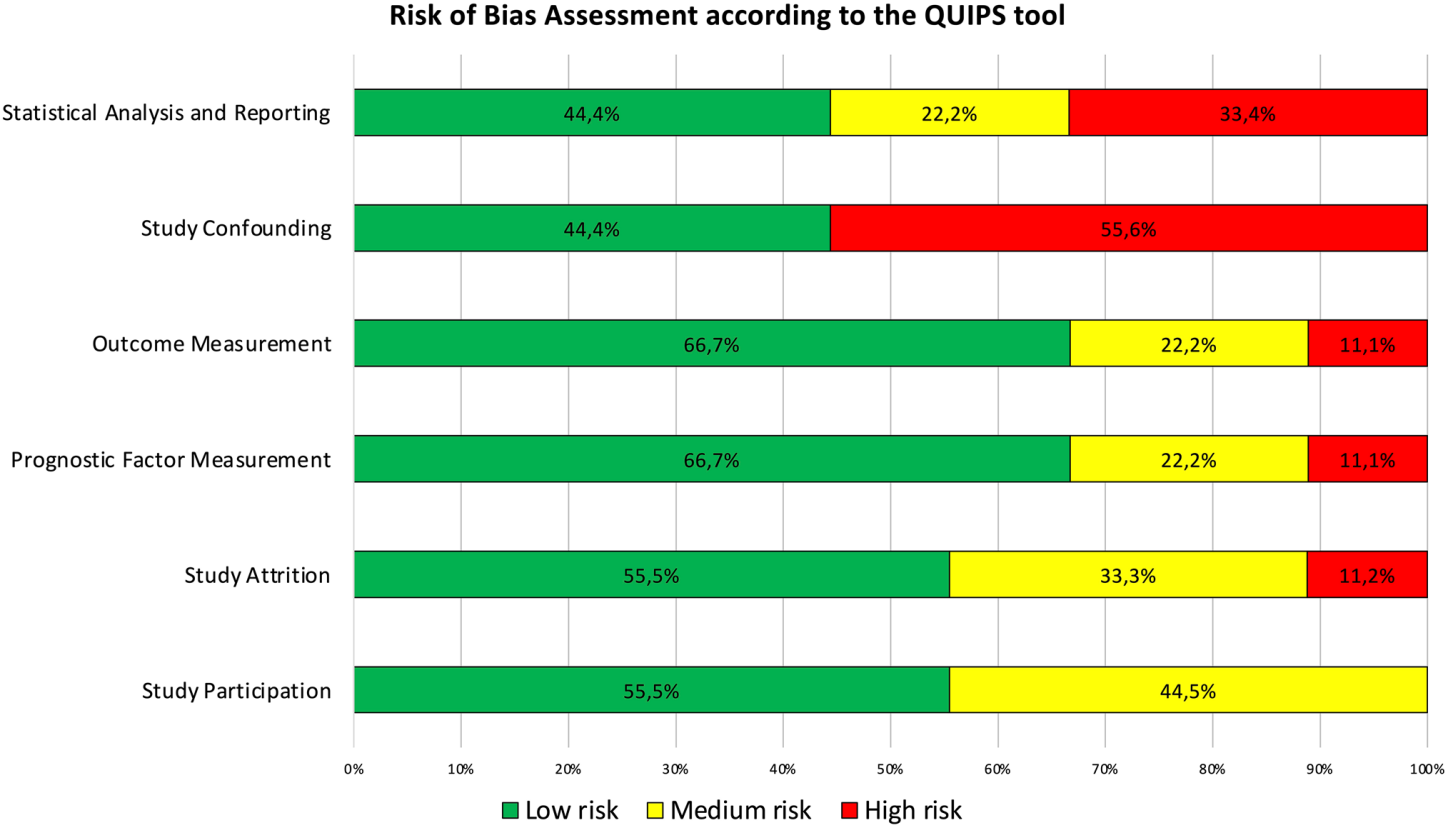
Graphical overview of the overall risk of bias judgements for the studies included in the review according to the Quality In Prognosis Studies (QUIPS) tool

Overall, eight studies reported a study population and involved a total of 159,136 patients (Table 1). Four studies performed a cost-analysis of a RCC screening program; three of these included a specific study population (Table 2) and one study was focused on a detailed cost analysis based on a decision model evaluating screening in asymptomatic individuals using focused US. Risk-of-bias assessment according to the QUIPS tool [20] is shown in Supplementary Table 1 and Fig. 2; the study confounding domain had the highest proportion of studies with high risk of bias (55.6%). The overall quality of evidence according to GRADE was low.

### Qualitative evidence synthesis

A detailed overview of the results of the studies assessing the benefits and harms of available RCC screening programs reporting a clear target population is shown in Table 1. Regarding the imaging modality for RCC screening, five studies employed ultrasound (US), one computed tomography (CT) scans, one positron emission tomography (PET), and one both CT scan and US.

Ono et al. [21] investigated the detection rate of a variety of cancers using whole-body PET for screening 3426 asymptomatic individuals, focusing on their clinical and pathological stages. Overall, the authors found one stage IV RCC in the PET positive group, while one stage I RCC and one stage III RCC in the PET negative group.

Malaeb et al. [22] investigated the power of US for screening 6678 consecutive patients in conjunction with the Aneurysm Detection and Management study. Overall, 817 (12.3%) renal anomalies were found, including a solid renal mass in 22 (0.32%) patients. Confirmatory CT or MRI was used in the 22 patients with solid renal masses. Histopathological confirmation after surgery could only be obtained in 15 RCC as five patients were lost during the follow-up and two were poor surgical candidates. The tumors were staged pT1 in 10 cases and pT3 in five cases; of the latter, two patients had a node positive disease and one had metastases at diagnosis. The histological classification included 13 cases of clear cell carcinoma, one of granular tumor and one of papillary tumor.

Mizuma et al. [23] investigated the findings of the first sonographic screening of 16,024 healthy subjects to assess the validity of US-based screening for abdominal cancers in an asymptomatic population. Four patients were diagnosed with RCC through the screening program and one was missed by US. All patients diagnosed with RCC by screening US underwent curable resection; the only case that was missed could not undergo surgery, because RCC presented with a bone metastasis 3 months later.

Haliloglu et al. [24] retrospectively reviewed the reports of 18.686 consecutive urinary US examinations of 18 203 patients were evaluated. 35 of the remaining 74 patients were diagnosed as angiomyolipoma by US, and confirmed by CT. Three renal masses that could not be classified by US were proved to be benign with other imaging techniques.

Thirty-six of the 74 patients with preoperative diagnosis of RCC and in one of them two pulmonary nodules were detected. After undergoing surgery, the renal masses underwent histopathological examination. Only the Fuhrman grading system of the RCC was reported in the study: four patients (11.2%) were classified as grade I, 19 patients (52.7%) were grade II, and 13 patients (36.1%) were grade III; no grade IV were reported.

Tsuboi et al. [25] analyzed 60,604 subjects undergoing a general health checkup employing US.

Of these, 97 were diagnosed as having a renal tumor or suspected renal tumor by transabdominal US screening and underwent re-examination by US or CT scan. Twenty-four patients (4 men and 20 women) were diagnosed as having AML, with a fatty component detected within the mass by plain CT, and two patients as having renal calcification. Contrast CT enabled diagnosis of a renal cyst in two patients and a renal tumor in 14 patients (nine men and five women). All 14 patients diagnosed as having RCC except one underwent nephrectomy, which gave pathologic evidence supporting the diagnosis of RCC. The remaining one patient refused to undergo the operation and was then followed up at regular intervals. No pathological report was shown in the study.

Filipas et al. [26] reported the results form a screening program of 9959 volunteers participated in the screening program in the first year and of these participants, 79% returned for re-examination in the second year. Thirteen (0.1%) subjects were found to have a renal mass, of which nine were RCC, one was leiomyoma, one a oncocytoma, and two were followed by imaging without undergoing surgery. RCC stage was reported: one T1N0M0, five T2N0M0, one pT3bN0M0, and two M1 (T3bN1 and T3bN2).

Mitchell et al. [27]m reported in a single-center study a population of 11,932 healthy asymptomatic subjects undergoing electron beam CT scan and a diagnosis of solid renal tumor was made for 26 patients and underwent resection of the renal mass. Overall, Nineteen renal cell carcinomas, three oncocytomas, two angiomyolipomas (AML), and one cystic nephroma were identified. Of the 22 classifiable tumors, 20 were T1N0M0, 1 was T2N0M0, and 1 was T3aN0M0. One patient was found to have adrenal hemorrhage and thrombosis without renal pathology. One patient died of surgical complications. Twenty-five patients are clinically well and without evidence of recurrent disease at 1–41 months (mean 17 months) postoperatively.

Feldstein et al. [28] reported 32,310 healthy subjected undergoing an executive health program employing both US and CT scan. 18 RCCs were detected and of these, 13 (72%) were detected by the screening program and five (28%) were missed. Of the detected RCC, 12 were T1N0M0 and 1 was T2N0M0; of the undetected RCC, one T1N0M0, one T2N0M0, one T3aN0M0, and two were M1 (T1Nx and T2N0).

Overall, the prevalence of renal masses/RCC diagnosed by imaging among the screened individuals ranged between 0.02% and 0.22% across the included studies (Table 1); of note, the prevalence of disease was inherently associated with the socio-demographic characteristics of the subjects included in the screening programs.

### Cost-analysis of RCC screening programs

A detailed overview of the results of the four studies assessing the costs of RCC screening programs is reported in Table 2.

Malaeb et al. [22] presented a limited cost analysis as not comprehensive of all expenses. Cost for US and CT with contrast medium were $107 and $337, respectively. However, they did not include several cost items, such as the radiologist’s fee, the office visits, loss of work and patient anxiety. At these rates the minimum cost of detecting one RCC was $32,480, which does not include additional studies derived from false-positive results requiring abdominal CT or MRI.

Mizuma et al. [23] reported a screening program comprehending 16,024 subjects undergoing US examinations; 762 patients underwent further diagnostic workup to detect 11 RCCs. Assuming a cost of $200 for US or subsequent testing, the expenditure was $3,357,200 for a total of 16,786 examinations and $305,200 for each screening-detected RCC.

Rossi et al. [12] conducted a study to determine whether current evidence suggests that screening is potentially cost-effective and, if so, in which age/gender groups. A decision model was developed evaluating screening in asymptomatic individuals in the UK, adopting a National Health Service perspective. The authors assessed the potential benefit and cost of a single focused renal US scan compared with standard of care (no screening). A comprehensive cost-analysis per gender and age groups was performed, proving that cost-effectiveness improves as the prevalence of RCC increases and the cost of US decreases. Overall, given a prevalence of RCC of 0.34% (0.18–0.54%), screening 60-year-old men resulted in an incremental cost-effectiveness ratio (ICER) of £18,092/quality-adjusted life years (QALY) (€22,843/QALY). Given a prevalence of RCC of 0.16% (0.08–0.25%), screening 60-year-old women resulted in an ICER of £37 327/ QALY (€47 129/QALY). In the male population, the authors reported a RCC prevalence of 0.14%, 0.23%, and 0.34% for patients > 40, > 50, and > 60 years, respectively, with an incremental cost for the health system of £47.06, £45.69, and £44.55, respectively. Similarly, but reflecting the lower prevalence, in the female population they reported a RCC prevalence of 0.07%, 0.09%, and 0.16% for patients > 40, > 50, and > 60 years, respectively, with an incremental cost of £47.61, £46.99, and £46.56. The authors concluded that current evidence suggests that one-off screening of 60-year-old men is potentially cost-effective and that further research into this topic would be of value to society.

Finally, Filipas et al. [26] evaluated cost-effectiveness as a critical issue for evaluating any screening program. They evaluated renal US as widely available and low cost (US $6 per investigation) favoring its use as a potential screening tool for RCC. However, the study stated that the low incidence of RCC compared with other screened cancers raises doubts about the economic benefit of screening a population of ≥ 40 years. In addition, no comprehensive evaluation has been performed as many expenses have not been investigated (i.e., cost of treatment of metastatic RCC and the prevalence of incurable disease).

### Current clinical trials on RCC screening

Only one clinical trial on RCC screening is currently ongoing (Yorkshire Kidney Screening Trial [YKST); NCT05005195), sponsored by the University of Leeds in U.K. This is a non-randomized open label feasibility study for a RCC screening program in patients enrolled in the Yorkshire Lung Screening Trial that offers subjects at high risk for lung cancer aged 55–80 a CT scan as part of a lung health check. The study started on May 10th, 2021 and the estimated tudy completion date is February 28th, 2023. To perform a correct RCC screening, additional low-dose abdominal CT scan slices are performed to achieve a correct visualization of the kidney. Primary Outcome Measures include: the proportion of individuals who take up the offer of an abdominal CT scan; the acceptability of the combined lung and RCC screening approach to participants by non-contrast CT scanning; the acceptability of the combined lung and RCC screening approach to healthcare professionals; and the additional time required for the combined screening approach, including the time to provide information, consent participants, and perform the lengthier CT scan, the time needed by radiologists for reporting the CT scans, and the additional time to review abdominal CT findings. Secondary outcomes include the proportion of participants found to have an RCC to provide an estimate of the prevalence of RCC at non-contrast CT screening in 55–80 year smokers and ex-smokers; the stage distribution of all RCCs identified; the proportion of participants found to have incidental renal findings (cysts, anatomical variants) on non-contrast CT scanning; and the proportion of participants with non-renal findings (i.e., abdominal aortic aneurysms, pancreatic and liver lesions on non-contrast CT scanning).

## Discussion

As no pre­malignant conditions can be identified and treated in a timely fashion before development of RCC, the aim of a RCC screening program is still to reduce deaths by the identification of tumors at an early and treatable stage [15]. In this systematic review we provided an updated summary on the available evidence on the currently available screening programs for RCC, focusing on their potential clinical impact and cost-effectiveness.

After decades of research on screening for RCC, a number of studies and clinical trials are now attempting to redesign the concept of RCC early detection [15]. The rationale behind a screening program for RCC is clear, given the high cancer-specific mortality, the increase prevalence of established RCC risk factors in the general population and the adverse consequences of late diagnosis and treatment at more advanced stages [29]. Notably, there is an increasing interest within the international Urology Community in evaluating the optimal strategy for earlier diagnosis of this ‘silent’ cancer, which is largely curable if identified at an early stage; this priority has also been recognized by patients with kidney cancer, caregivers and expert clinicians [30, 31]. In fact, the epidemiologic signature of the disease (i.e., increasing incidence, high proportion of asymptomatic individuals at diagnosis and high mortality rate) allows RCC to fulfil many of the Wilson and Jungner criteria for suitability for screening [32], although a number of key uncertainties still remain and require further research [15, 33]. In addition, while the “preclinical period” (defined as the time during which an individual has RCC but has not yet received a diagnosis) and consequently the time during which a screening program could be effective is estimated to be between 3.7 and 5.8 years in an asymptomatic screening population, whether detecting RCC during the preclinical period is ultimately beneficial is still controversial [2, 15].

The prevalence of asymptomatic RCC in previous studies including subjects receiving CT screening for coronary artery disease, lung cancer, colon cancer or as self-referred screenees ranged from 0.11 to 0.76% (pooled prevalence of 0.21%; 95% CI, 0.14–0.28%) [34]. Interestingly, the prevalence of renal masses and RCC detected in studies from Western Countries (Europe and North America) are more than double those in studies from Asia (0.17% versus 0.06%, respectively) [35]. These finding not only highlight a non-negligible variability in the estimates of RCC prevalence in asymptomatic individuals across Countries, but also underline that the concept of screening for “(small) renal masses” (of undetermined nature) does not necessarily equal the concept of screening for histologically defined RCC. To provide an overview of the available studies addressing the clinical utility of *RCC* screening programs, in this work we explicitly excluded from the analysis studies reporting on screening programs for “renal masses” without histopathological confirmation of RCC nature.

The ideal screening modality for RCC is yet to be elucidated. A recent systematic review on liquid biomarkers and innovative imaging modalities for RCC diagnosis found that none of the proposed tests were ready for prime time; as such, despite the promising role of miRNAs, metabolites and CT- or MRI-based radiomics features coupled with machine-learning algorithms, the current evidence appears premature to recommend integration of noninvasive diagnostic modalities in routine clinical practice [14]. For a standalone RCC screening program, US of the kidney has been proposed as an effective option. Our review found that the most commonly investigated screening modalities were indeed focused renal US followed by CT scan. US holds the advantages of being a widely available modality all over the world, to be highly cost-effective, and to be non-invasive in terms of radiations; nonetheless, its limitations is mostly linked to the operator-dependency of the technique (that can be performed by radiologists or urologists depending on several logistical issues within organizations and Countries) as well as to the variety of patient-related factors potentially impacting on US quality (such as obesity). Ultrasound RCC detection rates also depend on the size of renal lesions: while it enables the detection of 85–100% tumors > 3 cm in size, only 67–82% of tumors 2–3 cm in size can be diagnosed [36, 37]. Therefore, ultrasound screening for RCC has the potential to lead to false-negative results in masses < 3 cm in size. Finally, detection of a (small) renal mass at renal US does not necessarily mean detection of RCC, due to the non-negligible rate of benign renal masses in such patient populations [38]. On the contrary, abdominal contrast­enhanced CT is still the gold­standard method of detecting a renal mass. CT scan yields the advantage of being objective and more precise in depicting abdominal organs; at the same time, despite the high sensibility and specificity, CT scan leads to higher costs, invasiveness, and risk of adverse events. As such, CT might not be appropriate for national screening due to several caveats, including the relatively low prevalence of renal masses in the general population, the high cost, and potential risks [15]. Yet, combining RCC CT scanning with other CT­scan­based health check programs might be an option to increase the value and cost-effectiveness of such screening programs; this concept is indeed currently being explored in the Yorkshire Kidney Screening Trial (NCT05005195). There are a few studies exploring RCC screening in conjunction with other screening programs, such as abdominal aortic aneurism [22] or lung cancer [39], with the aim to decrease costs, invasiveness and to increase the diagnosis of a pathological finding, since these diseases have a low prevalence in the healthy population.

In our review, the overall prevalence of renal masses/RCC diagnosed by imaging ranged between 0.02 and 0.22% across the included studies (Table 1). It is important to highlight that while the incidence of RCC increases with age, screening elderly individuals with potentially higher competing risks of death might not be beneficial. A recently published analysis of US showed that screening men aged 50–60 years was the most cost­effective approach [12]. Cost­effectiveness modelling is also highly sensitive to the prevalence of RCC (Table 2) [12]: targeted screening of higher risk individuals, selected from the general population by means of established risk-stratification tools [40] is likely to be the most cost­effective strategy [41]. Any RCC screening program should indeed also aim to minimize potential harms to individual subjects and to the whole society, including worsening of quality of life, emotional distress, and incidental overdiagnosis (and overtreatment) of benign or slowly growing indolent renal masses; to achieve this goal, refinement of current decision-making schemes for patients with localized renal masses (including the role of renal tumor biopsy) are urgently warranted [42]. Screening programs for RCC will be effective only if subjects are willing to undergo screening. In this regard, in a recent online population‐based survey aiming to explore attitudes towards kidney cancer screening and factors influencing intention to attend a future screening programs, most participants were ‘very likely’ or ‘likely’ to undergo screening tests for RCC using urine-, blood-, US- or low‐dose CT-based tests, both within a RCC-specific program and in conjunction with lung cancer screening [43]. These findings support the ongoing research into RCC screening tests [15]: based on the available evidence, specific combinations of serum or urinary miRNAs, as well as other biomarkers, such as serum (or, less frequently, urinary) proteins, enzymes, metabolites, GAGs, and cell-free DNA, appear promising for discrimination between healthy controls and patients with histologically confirmed RCC. One clinical trial (NCT02923284) evaluated two biomarkers (urinary AQP1 and PLIN2) as a low-cost screening method to discriminate benign renal masses and patients with RCC from healthy controls [14]. Further research is needed to validate these biomarkers as efficient and cost-effective screening tools at a population-level and in clinical practice.

Our systematic review is not devoid of limitations. First, despite a rigorous review process and a well-defined PICO framework, our search strategy might have not retrieved all potentially relevant articles on RCC screening. Moreover, we limited our search to the English-language literature. Of note, we could not analyze the differential impact of available screening programs on the detection of indeterminate renal masses *versus* pathologically confirmed RCC, mainly due to limitations in the design of available studies. For the same reason, we could not analyze in detail the cost-effectiveness of all available RCC screening programs, nor perform a formal quantitative analysis from the studies included in the review.

## Conclusions

Despite an increasing interest from patients and clinicians during the last decades, our systematic review found a relative lack of studies reporting the efficacy and cost-effectiveness of well-structured screening programs for RCC. There is no high-quality evidence to show that screening asymptomatic individuals by any biomarker- or imaging-based test would reduce RCC morbidity and/or mortality. Moreover, the optimal modality for RCC screening is still controversial. Targeting high-risk individuals and/or combining detection of RCC with other health checks such as lung cancer screening represent pragmatic options to improve the cost-effectiveness and reduce the potential harms of RCC screening. While waiting the results of prospective clinical trials, further research is needed to develop and validate accurate risk prediction models for RCC, define the most cost-effective screening population, explore the acceptability of a screening program in the population, and assess whether RCC screening might lead to a survival benefit without further increasing overdiagnosis and overtreatment of localized renal masses.

## Supplementary Information

Below is the link to the electronic supplementary material.

Supplementary file1 (DOCX 24 kb)

Supplementary file2 (DOCX 17 kb)

Supplementary file3 (DOCX 31 kb)
