# Supplementary material for: Screening programs for renal cell carcinoma: a systematic review by the EAU young academic urologists renal cancer working group
Source: World J Urol. 2022 Apr 1;41(4):929–40. doi: 10.1007/s00345-022-03993-6 (PMC10160199; doi:10.1007/s00345-022-03993-6)
Supplement: Supplementary file 1 — Supplementary file1 (DOCX 24 kb) [file 345_2022_3993_MOESM1_ESM.docx]

**Screening Programs for Renal Cell Carcinoma:**

**a Systematic Review by the EAU Young Academic Urologists Renal Cancer Working Group.**

Pietro Diana^1,2^*, Tobias Klatte^3,4^, Daniele Amparore^5,6^, Riccardo Bertolo^6,7^, Umberto Carbonara^6,8^, Selcuk Erdem^6,9^, Alexandre Ingels^6,10,11^, Onder Kara^6,12^, Laura Marandino^6,13^, Michele Marchioni^6,14,15^, Stijn Muselaers^6,16^, Nicola Pavan^6,17^, Angela Pecoraro^5,6^, Alessio Pecoraro^18^, Eduard Roussel^6,19^, and Riccardo Campi^6,18,20^*

*on behalf of the European Association of Urology (EAU) Young Academic Urologists (YAU) Renal Cancer group.*

**Appendix: literature search strategy**

1. **PICO framework**

- (P): adult (>18 years) healthy subjects or adult individuals at higher risk of developing renal cell carcinoma (RCC) based on established risk factors (age, male gender, family history, smoking, obesity, diabetes, hypertension) with no prior history of RCC (or prior imaging showing a renal mass) and no prior history of diseases increasing the risk of RCC (including genetic syndromes);
- (I): any screening intervention (opportunistic or population screening), including any type of medical test (liquid biomarkers, non-invasive imaging, renal biopsy);
- (C): either comparative or non-comparative studies;
- (O): cost-effectiveness of the screening program (detection rate of histologically confirmed RCC vs costs of the screening program). Studies assessing the impact of screening programs on the detection of renal masses of undetermined nature will be excluded

1. **Literature Search strategy (for the MEDLINE [via PubMed] database)**
   1. ***Concept of RCC***

Search: **((((renal cell carcinoma) OR (renal neoplasm[MeSH Terms])) OR (renal cancer)) OR (kidney cancer)) OR (kidney neoplasm[MeSH Terms])** Sort by: **Most Recent**

"carcinoma, renal cell"[MeSH Terms] OR ("carcinoma"[All Fields] AND "renal"[All Fields] AND "cell"[All Fields]) OR "renal cell carcinoma"[All Fields] OR ("renal"[All Fields] AND "cell"[All Fields] AND "carcinoma"[All Fields]) OR "kidney neoplasms"[MeSH Terms] OR ("kidney neoplasms"[MeSH Terms] OR ("kidney"[All Fields] AND "neoplasms"[All Fields]) OR "kidney neoplasms"[All Fields] OR ("renal"[All Fields] AND "cancer"[All Fields]) OR "renal cancer"[All Fields]) OR ("kidney neoplasms"[MeSH Terms] OR ("kidney"[All Fields] AND "neoplasms"[All Fields]) OR "kidney neoplasms"[All Fields] OR ("kidney"[All Fields] AND "cancer"[All Fields]) OR "kidney cancer"[All Fields]) OR "kidney neoplasms"[MeSH Terms]

**Translations**

**renal cell carcinoma:** "carcinoma, renal cell"[MeSH Terms] OR ("carcinoma"[All Fields] AND "renal"[All Fields] AND "cell"[All Fields]) OR "renal cell carcinoma"[All Fields] OR ("renal"[All Fields] AND "cell"[All Fields] AND "carcinoma"[All Fields])

**renal neoplasm[MeSH Terms]:** "kidney neoplasms"[MeSH Terms]

**renal cancer:** "kidney neoplasms"[MeSH Terms] OR ("kidney"[All Fields] AND "neoplasms"[All Fields]) OR "kidney neoplasms"[All Fields] OR ("renal"[All Fields] AND "cancer"[All Fields]) OR "renal cancer"[All Fields]

**kidney cancer:** "kidney neoplasms"[MeSH Terms] OR ("kidney"[All Fields] AND "neoplasms"[All Fields]) OR "kidney neoplasms"[All Fields] OR ("kidney"[All Fields] AND "cancer"[All Fields]) OR "kidney cancer"[All Fields]

**kidney neoplasm[MeSH Terms]:** "kidney neoplasms"[MeSH Terms]

- 1. ***Concept of screening***

Search: **(screening) OR (cancer screening[MeSH Terms])** Sort by: **Most Recent**

"diagnosis"[MeSH Subheading] OR "diagnosis"[All Fields] OR "screening"[All Fields] OR "mass screening"[MeSH Terms] OR ("mass"[All Fields] AND "screening"[All Fields]) OR "mass screening"[All Fields] OR "early detection of cancer"[MeSH Terms] OR ("early"[All Fields] AND "detection"[All Fields] AND "cancer"[All Fields]) OR "early detection of cancer"[All Fields] OR "screen"[All Fields] OR "screenings"[All Fields] OR "screened"[All Fields] OR "screens"[All Fields] OR "early detection of cancer"[MeSH Terms]

**Translations**

**screening:** "diagnosis"[Subheading] OR "diagnosis"[All Fields] OR "screening"[All Fields] OR "mass screening"[MeSH Terms] OR ("mass"[All Fields] AND "screening"[All Fields]) OR "mass screening"[All Fields] OR "early detection of cancer"[MeSH Terms] OR ("early"[All Fields] AND "detection"[All Fields] AND "cancer"[All Fields]) OR "early detection of cancer"[All Fields] OR "screen"[All Fields] OR "screenings"[All Fields] OR "screened"[All Fields] OR "screens"[All Fields]

**cancer screening[MeSH Terms]:** "early detection of cancer"[MeSH Terms]

- 1. ***Concept of RCC & Screening***

Search: **((screening) OR (cancer screening[MeSH Terms])) AND (((((renal cell carcinoma) OR (renal neoplasm[MeSH Terms])) OR (renal cancer)) OR (kidney cancer)) OR (kidney neoplasm[MeSH Terms]))** Sort by: **Most Recent**

("diagnosis"[MeSH Subheading] OR "diagnosis"[All Fields] OR "screening"[All Fields] OR "mass screening"[MeSH Terms] OR ("mass"[All Fields] AND "screening"[All Fields]) OR "mass screening"[All Fields] OR "early detection of cancer"[MeSH Terms] OR ("early"[All Fields] AND "detection"[All Fields] AND "cancer"[All Fields]) OR "early detection of cancer"[All Fields] OR "screen"[All Fields] OR "screenings"[All Fields] OR "screened"[All Fields] OR "screens"[All Fields] OR "early detection of cancer"[MeSH Terms]) AND ("carcinoma, renal cell"[MeSH Terms] OR ("carcinoma"[All Fields] AND "renal"[All Fields] AND "cell"[All Fields]) OR "renal cell carcinoma"[All Fields] OR ("renal"[All Fields] AND "cell"[All Fields] AND "carcinoma"[All Fields]) OR "kidney neoplasms"[MeSH Terms] OR ("kidney neoplasms"[MeSH Terms] OR ("kidney"[All Fields] AND "neoplasms"[All Fields]) OR "kidney neoplasms"[All Fields] OR ("renal"[All Fields] AND "cancer"[All Fields]) OR "renal cancer"[All Fields]) OR ("kidney neoplasms"[MeSH Terms] OR ("kidney"[All Fields] AND "neoplasms"[All Fields]) OR "kidney neoplasms"[All Fields] OR ("kidney"[All Fields] AND "cancer"[All Fields]) OR "kidney cancer"[All Fields]) OR "kidney neoplasms"[MeSH Terms])

**Translations**

**screening:** "diagnosis"[Subheading] OR "diagnosis"[All Fields] OR "screening"[All Fields] OR "mass screening"[MeSH Terms] OR ("mass"[All Fields] AND "screening"[All Fields]) OR "mass screening"[All Fields] OR "early detection of cancer"[MeSH Terms] OR ("early"[All Fields] AND "detection"[All Fields] AND "cancer"[All Fields]) OR "early detection of cancer"[All Fields] OR "screen"[All Fields] OR "screenings"[All Fields] OR "screened"[All Fields] OR "screens"[All Fields]

**cancer screening[MeSH Terms]:** "early detection of cancer"[MeSH Terms]

**renal cell carcinoma:** "carcinoma, renal cell"[MeSH Terms] OR ("carcinoma"[All Fields] AND "renal"[All Fields] AND "cell"[All Fields]) OR "renal cell carcinoma"[All Fields] OR ("renal"[All Fields] AND "cell"[All Fields] AND "carcinoma"[All Fields])

**renal neoplasm[MeSH Terms]:** "kidney neoplasms"[MeSH Terms]

**renal cancer:** "kidney neoplasms"[MeSH Terms] OR ("kidney"[All Fields] AND "neoplasms"[All Fields]) OR "kidney neoplasms"[All Fields] OR ("renal"[All Fields] AND "cancer"[All Fields]) OR "renal cancer"[All Fields]

**kidney cancer:** "kidney neoplasms"[MeSH Terms] OR ("kidney"[All Fields] AND "neoplasms"[All Fields]) OR "kidney neoplasms"[All Fields] OR ("kidney"[All Fields] AND "cancer"[All Fields]) OR "kidney cancer"[All Fields]

**kidney neoplasm[MeSH Terms]:** "kidney neoplasms"[MeSH Terms]

- 1. ***English-language literature (filter)***

Search: **((screening) OR (cancer screening[MeSH Terms])) AND (((((renal cell carcinoma) OR (renal neoplasm[MeSH Terms])) OR (renal cancer)) OR (kidney cancer)) OR (kidney neoplasm[MeSH Terms]))** Filters: **English** Sort by: **Most Recent**

<https://pubmed.ncbi.nlm.nih.gov/?term=%28%28screening%29+OR+%28cancer+screening%5BMeSH+Terms%5D%29%29+AND+%28%28%28%28%28renal+cell+carcinoma%29+OR+%28renal+neoplasm%5BMeSH+Terms%5D%29%29+OR+%28renal+cancer%29%29+OR+%28kidney+cancer%29%29+OR+%28kidney+neoplasm%5BMeSH+Terms%5D%29%29&filter=lang.english&sort=date>

- 1. ***Literature published after 2000***

Search: **((screening) OR (cancer screening[MeSH Terms])) AND (((((renal cell carcinoma) OR (renal neoplasm[MeSH Terms])) OR (renal cancer)) OR (kidney cancer)) OR (kidney neoplasm[MeSH Terms]))** Filters: **English, from 2000/1/1 - 2021/11/14** Sort by: **Most Recent**

31561 records

<https://pubmed.ncbi.nlm.nih.gov/?term=%28%28screening%29+OR+%28cancer+screening%5BMeSH+Terms%5D%29%29+AND+%28%28%28%28%28renal+cell+carcinoma%29+OR+%28renal+neoplasm%5BMeSH+Terms%5D%29%29+OR+%28renal+cancer%29%29+OR+%28kidney+cancer%29%29+OR+%28kidney+neoplasm%5BMeSH+Terms%5D%29%29&filter=dates.2000%2F1%2F1-2021%2F11%2F14&filter=lang.english&sort=date>
