# Supplementary material for: Screening programs for renal cell carcinoma: a systematic review by the EAU young academic urologists renal cancer working group
Source: World J Urol. 2022 Apr 1;41(4):929–40. doi: 10.1007/s00345-022-03993-6 (PMC10160199; doi:10.1007/s00345-022-03993-6)
Supplement: Supplementary file 2 — Supplementary file2 (DOCX 17 kb) [file 345_2022_3993_MOESM2_ESM.docx]

**Table.** Risk of bias assessment within individual studies according to the Quality In Prognosis Studies (QUIPS) tool.

| **Number** | **Report** | **Reporting and Quality in Prognosis Studies (QUIPS) tool -** ☺Low Risk; 〇 Moderate Risk; ☹High Risk | | | | | |
| --- | --- | --- | --- | --- | --- | --- | --- |
|  |  | **QUIPS tool: domain 1**  (study participation) | **QUIPS tool: domain 2**  (study attrition) | **QUIPS tool: domain 3** (prognostic factor measurement) | **QUIPS tool: domain 4** (outcome measurement) | **QUIPS tool: domain 5** (study confounding) | **QUIPS tool: domain 6** (statistical analysis and reporting) |
| 1 | Ono et al. | 〇 | ☺ | ☺ | ☺ | ☹ | ☹ |
| 2 | Malaeb et al. | ☺ | ☺ | ☺ | ☺ | ☺ | ☺ |
| 3 | Mizuma et al. | ☺ | 〇 | 〇 | 〇 | ☹ | ☹ |
| 4 | Rossi et al. | 〇 | ☹ | ☺ | ☺ | ☺ | ☺ |
| 5 | Haliloglu et al. | 〇 | 〇 | 〇 | ☺ | ☹ | ☹ |
| 6 | Tsuboi et al. | 〇 | 〇 | ☹ | ☹ | ☹ | 〇 |
| 7 | Filipas et al. | ☺ | ☺ | ☺ | ☺ | ☺ | ☺ |
| 8 | Mitchell et al. | ☺ | ☺ | ☺ | 〇 | ☹ | 〇 |
| 9 | Feldestein et al. | ☺ | ☺ | ☺ | ☺ | ☺ | ☺ |
